# Inorganic arsenic causes fatty liver and interacts with ethanol to cause alcoholic liver disease in zebrafish

**DOI:** 10.1242/dmm.031575

**Published:** 2018-02-01

**Authors:** Kathryn Bambino, Chi Zhang, Christine Austin, Chitra Amarasiriwardena, Manish Arora, Jaime Chu, Kirsten C. Sadler

**Affiliations:** 1 Department of Environmental Medicine and Public Health, Icahn School of Medicine at Mount Sinai, New York, New York 10029, USA; 2 Program in Biology, New York University Abu Dhabi, Saadiyat Island Campus, PO Box 129188 Abu Dhabi, United Arab Emirates; 3 Department of Pediatrics, Division of Pediatric Hepatology, Icahn School of Medicine at Mount Sinai, New York, New York 10029, USA

**Keywords:** Arsenic, Ethanol, Fatty liver disease, Environmental exposure

## Abstract

The rapid increase in fatty liver disease (FLD) incidence is attributed largely to genetic and lifestyle factors; however, environmental toxicants are a frequently overlooked factor that can modify the effects of more common causes of FLD. Chronic exposure to inorganic arsenic (iAs) is associated with liver disease in humans and animal models, but neither the mechanism of action nor the combinatorial interaction with other disease-causing factors has been fully investigated. Here, we examined the contribution of iAs to FLD using zebrafish and tested the interaction with ethanol to cause alcoholic liver disease (ALD). We report that zebrafish exposed to iAs throughout development developed specific phenotypes beginning at 4 days post-fertilization (dpf), including the development of FLD in over 50% of larvae by 5 dpf. Comparative transcriptomic analysis of livers from larvae exposed to either iAs or ethanol revealed the oxidative stress response and the unfolded protein response (UPR) caused by endoplasmic reticulum (ER) stress as common pathways in both these models of FLD, suggesting that they target similar cellular processes. This was confirmed by our finding that arsenic is synthetically lethal with both ethanol and a well-characterized ER-stress-inducing agent (tunicamycin), suggesting that these exposures work together through UPR activation to cause iAs toxicity. Most significantly, combined exposure to sub-toxic concentrations of iAs and ethanol potentiated the expression of UPR-associated genes, cooperated to induce FLD, reduced the expression of *as3mt*, which encodes an arsenic-metabolizing enzyme, and significantly increased the concentration of iAs in the liver. This demonstrates that iAs exposure is sufficient to cause FLD and that low doses of iAs can potentiate the effects of ethanol to cause liver disease.

This article has an associated First Person interview with the first author of the paper.

## Abstract (precis)

**Summary:** Using zebrafish, the authors show that exposure to a common environmental contaminant, inorganic arsenic, increases the risk of alcoholic liver disease.

## INTRODUCTION

Fatty liver disease (FLD) is the most common liver pathology in the world (Levene and Goldin, 2012). The dramatic rise in incidence in the past several decades has prompted intense investigation into the biological basis for this observation. A high fat, high sugar diet (Adams and Lindor, 2007), genetic predisposition (Anstee and Day, 2015; Liu et al., 2013) and alcohol abuse (Magdaleno et al., 2017) are clear risk factors for FLD, but these risks alone do not account for the steep rise in FLD incidence, nor do they provide an explanation for all FLD cases. Epidemiological studies have shown that multiple environmental and anthropogenic toxicants cause liver disease in humans (Das et al., 2010; Islam et al., 2011; Mazumder, 2005; Santra et al., 1999), and work in rodents (Ditzel et al., 2016) and zebrafish (Cheng et al., 2016) have demonstrated a direct, causative relationship between some environmental toxicants and FLD (Al-Eryani et al., 2015; Wahlang et al., 2013). The combination of epidemiological and basic research on environmental toxicants and metabolic disease is rapidly advancing, yet the scope of the problem and the mechanisms of toxicity are not yet clear.

Chronic exposure to inorganic arsenic (iAs) is a worldwide public health concern as it is associated with a broad range of health problems (Centeno et al., 2002; Naujokas et al., 2013). iAs is a naturally occurring element in the earth's crust and both humans and wildlife are exposed to iAs through food and water. Estimates of over 100-million people worldwide are exposed to levels exceeding World Health Organization (WHO)-established limits (Adams et al., 2016; Farzan et al., 2016; Yang et al., 2009). The first prospective cohort study of people chronically exposed to arsenic has revealed an increase in all-cause and chronic disease mortalities (Ahsan et al., 2006; Argos et al., 2010). Notably, frequent co-morbidities of FLD, including diabetes (Kuo et al., 2015; Wang et al., 2014b), cardiovascular disease (Moon et al., 2013) and liver cancer (Wang et al., 2014a), are significantly associated with iAs exposure. A study in the arsenic-endemic regions of Bangladesh and West Bengal, India, where obesity and alcohol abuse are low, found that chronic exposure to iAs via drinking water is associated with liver damage and fibrosis (Das et al., 2012; Islam et al., 2011). This was confirmed by the finding of a high prevalence of FLD and other liver diseases in this same region (Das et al., 2010). Together, the epidemiological data suggests that arsenic is a liver toxicant in humans. Whether it can potentiate the effects of other causes of liver disease, such as alcohol abuse, remains to be investigated.

Work using animal models has demonstrated that iAs can cause FLD. In some mouse studies, chronic iAs exposure induces lipogenic gene expression in the liver (Adebayo et al., 2015) and FLD (Santra et al., 2000b). Strikingly, exposure to iAs *in utero* and post-weaning leads to adults that have a higher rate of FLD when fed a high-fat diet (Ditzel et al., 2016). In zebrafish, arsenic exposure in embryos causes widespread developmental defects (Adeyemi et al., 2015; Bambino and Chu, 2017; Li et al., 2009, 2012; Ma et al., 2015; McCollum et al., 2014; Wang et al., 2006), and exposing adult zebrafish to arsenic causes a range of gene and protein expression changes in the liver related to lipid metabolism (Carlson and Van Beneden, 2014; Hallauer et al., 2016; Li et al., 2016; Xu et al., 2013); one study reported FLD in adult zebrafish acutely exposed to iAs (Li et al., 2016). Thus, across species, iAs causes liver damage. These data indicate that iAs alone causes FLD and can also predispose to FLD susceptibility, and we propose that lower doses of iAs interact with more common risk factors to promote FLD.

The importance of iAs as a toxicant has generated significant interest in deciphering how iAs exposure causes disease. iAs metabolism via arsenic 3 methyltransferase (AS3MT) utilizes the same methyl donor that is used for DNA methylation (Hamdi et al., 2012; Thomas et al., 2004) and the iAs methylation reaction produces reactive oxygen species (ROS) (Jomova et al., 2011; Santra et al., 2000a; Shi et al., 2004; Xu et al., 2017). Thus, reduction in DNA methylation and increased oxidative stress are two leading theories for the mechanism of iAs toxicity. A third possibility is based on the finding that iAs impairs protein folding: iAs binds thiol groups, and it is well established that the basis for acute arsenic poisoning is iAs acting as a reducing agent for sulfhydryl groups in key metabolic enzymes (Hughes, 2002; Sattar et al., 2016). Similarly, at lower doses, iAs acts to reduce sulfhydryl groups on cysteine residues in nascent peptides, which prevents disulfide bond formation (Jacobson et al., 2012; Ramadan et al., 2009) and prevents accurate protein folding. In addition, ROS generated via arsenic metabolism can disrupt the redox balance required for disulfide bond formation and protein folding in the endoplasmic reticulum (ER). The finding that the unfolded protein response (UPR), the pathway induced by ER stress, is activated with iAs treatment of some cell types (Doudican et al., 2012; Weng et al., 2014) supports the hypothesis of ER stress as a mechanism for iAs-induced toxicity.

The UPR is a central pathway in FLD pathophysiology across species (Goessling and Sadler, 2015; Wang and Kaufman, 2014). Robust ER stress is sufficient to cause FLD in mice and zebrafish (Cinaroglu et al., 2011; Ji et al., 2011; Ozcan et al., 2004; Thakur et al., 2011; Vacaru et al., 2014; Yamamoto et al., 2010), and we have shown that activation of Atf6, a main upstream player in the UPR, is necessary and sufficient to cause FLD (Cinaroglu et al., 2011; Howarth et al., 2014). This provides a direct and mechanistic link between UPR activation and fatty liver. ROS generation from ethanol metabolism is a central mechanism of alcoholic liver disease (ALD) (Louvet and Mathurin, 2015). ROS alone can induce the UPR, and we (Tsedensodnom et al., 2013) and others (Lu and Cederbaum, 2008) have shown that the UPR activation and FLD caused by alcohol is mediated by ROS. Given that UPR activation is a central mechanism of ALD (Cinaroglu et al., 2011; Howarth et al., 2012, 2014), we hypothesize that other toxicants that disrupt ER function, such as iAs, could collaborate with ethanol to cause liver disease.

In this study, we use zebrafish to identify the mechanism by which iAs causes liver disease and to investigate whether iAs exposure interacts with ethanol to induce ALD. We found that iAs alone can cause FLD, and that sub-toxic concentrations of iAs and ethanol interact to induce the UPR and cause FLD. Transcriptomic analysis revealed that the ER- and oxidative-stress responses are activated in the liver of larvae treated with either iAs or ethanol, but, strikingly, although a few UPR target genes were common to both, these two toxicants induced unique UPR signatures. We found that larvae exposed to iAs are sensitized to ethanol, showing an increase in larval mortality and increased iAs concentration in the liver. Importantly, we show that iAs and ethanol interact to cause FLD. This provides evidence that environmental toxicants can modify the effect of more common risk factors for liver disease and thus may act as coordinating events in the multistep process of progressive liver disease (Browning and Horton, 2004; Day and James, 1998; Dowman et al., 2010).

## RESULTS

### Zebrafish are susceptible to arsenic toxicity

**Fig. 1.**
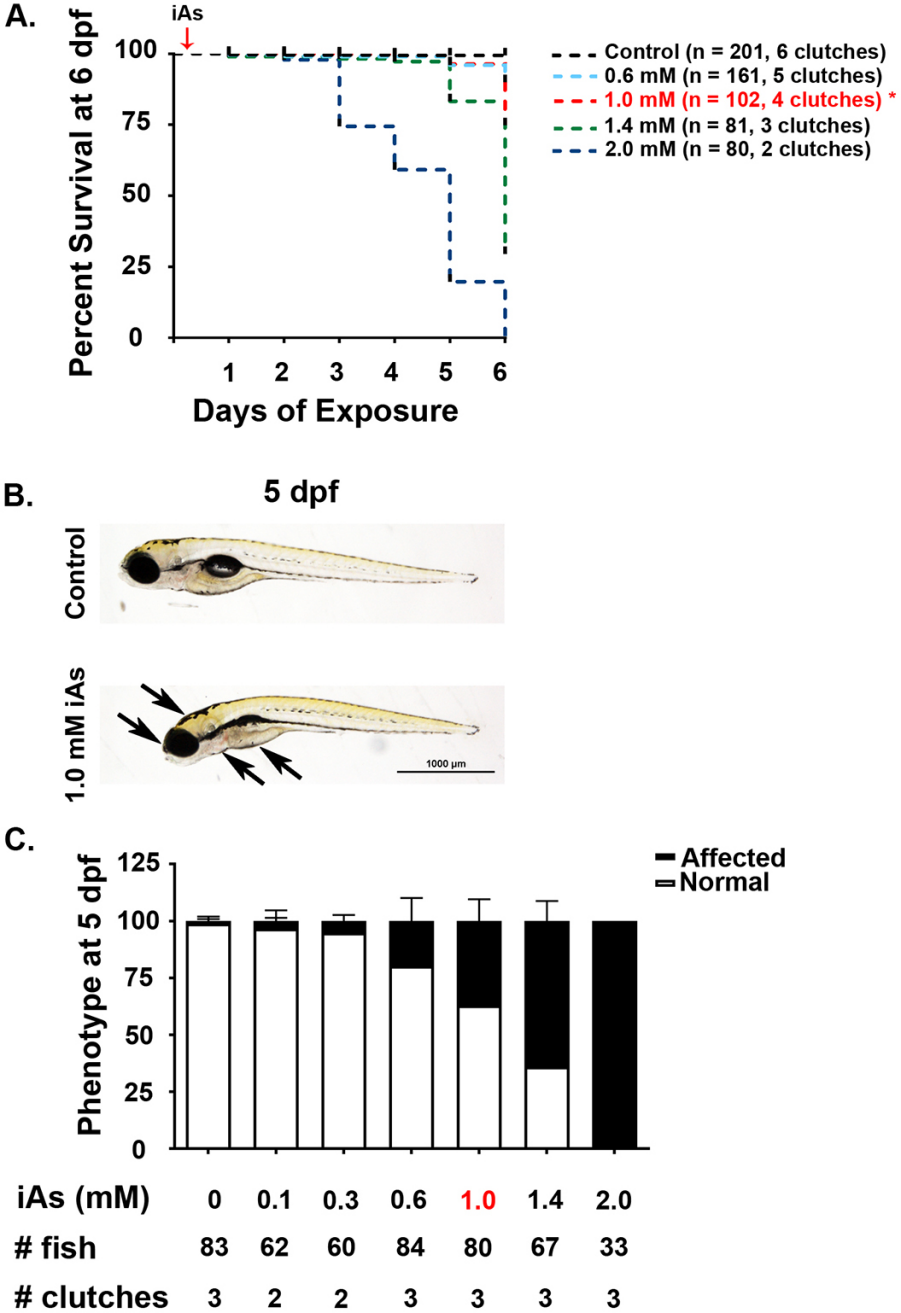
**Exposure to iAs is toxic to zebrafish embryos.** (A) Survival analysis of zebrafish treated with iAs from 4 hpf through 6 dpf. Fish were scored daily for mortality (*n*≥2 clutches, >80 embryos per condition, Table S1). Red arrow indicates addition of iAs. Concentration of iAs used throughout the manuscript is shown in red and indicated by an asterisk. (B) Bright-field images of representative wild-type control (top) and arsenic-exposed (bottom) 5 dpf zebrafish larvae. Arrows indicate arsenic-induced phenotypes, including shortened body length, edema, clustering of pigment cells, under-consumption of yolk, and a small head. (C) Exposure to increasing doses of iAs from 4 hpf to 5 dpf led to an accumulation of phenotypes. The proportion of surviving embryos at 5 dpf that were affected increased with increasing concentrations of iAs (*n*=2 clutches exposed to 0.1 mM or 0.3 mM, *n*=3 clutches exposed to 0.6 mM, 1.0 mM or 1.4 mM, >30 fish exposed per treatment condition, Table S1). hpf, hours post-fertilization; dpf, days post-fertilization; iAs, inorganic arsenic.

Multiple theories have been proposed to explain how chronic iAs causes disease; however, none have yet been conclusively identified. Studies using zebrafish to investigate the developmental toxicity of iAs (Li et al., 2009; Ma et al., 2015; Seok et al., 2007) and consequences of iAs exposure in adults (Carlson and Van Beneden, 2014; Hallauer et al., 2016; Lam et al., 2006; Xu et al., 2013) have established this as an excellent model for investigating the mechanism of iAs toxicity. We extended this work by establishing a protocol to expose zebrafish embryos to iAs throughout development at a dose that would not impair liver development, to allow analysis of liver disease in older larvae. Zebrafish embryos were exposed to a range of sodium (meta)arsenite (iAs) concentrations beginning at 4 hpf and monitored daily for mortality. Treatment with 2.0 mM (260 ppm) iAs resulted in death of all exposed fish by 6 dpf (*n*=2 clutches, 80 embryos per condition), whereas concentrations <1.0 mM were well tolerated during this time (Fig. 1A, Table S1). Using this data, we calculated the concentration at which 50% of exposed fish died by 6 dpf (LC_50_) to be 1.25 mM (Fig. S1A). Short-term exposure to 2 mM iAs prior to 48 hpf resulted in fewer developmental defects than longer exposures (Fig. S1B), suggesting that the effects of iAs exposure during development are cumulative. Exposure to the highest dose of iAs (2 mM) beginning at 72 or 96 hpf until 120 hpf also resulted in significant abnormalities and larval death (data not shown), raising the possibility that later developmental stages may also be susceptible; however, this remains an area for further exploration.

Embryos exposed to concentrations of iAs that caused minimal lethality by 5 dpf (i.e. <1.4 mM) developed a range of specific phenotypes by 5 dpf, including a shortened body length, edema, clustering of pigment cells, under-consumption of yolk and a small head (arrows, Fig. 1B). Of the larvae that survived exposure to 0.6, 1.0, 1.4 and 2.0 mM iAs until 5 dpf, 19.6, 37.1, 64.1 and 100%, respectively, displayed at least one of these phenotypes at 5 dpf (Fig. 1C and Fig. S1C). These data are consistent with other studies that show that iAs at concentrations of 1.0 mM and above caused developmental abnormalities, whereas 0.5 mM was well tolerated by developing zebrafish embryos (Li et al., 2009).

### Zebrafish metabolize iAs in the liver

**Fig. 2.**
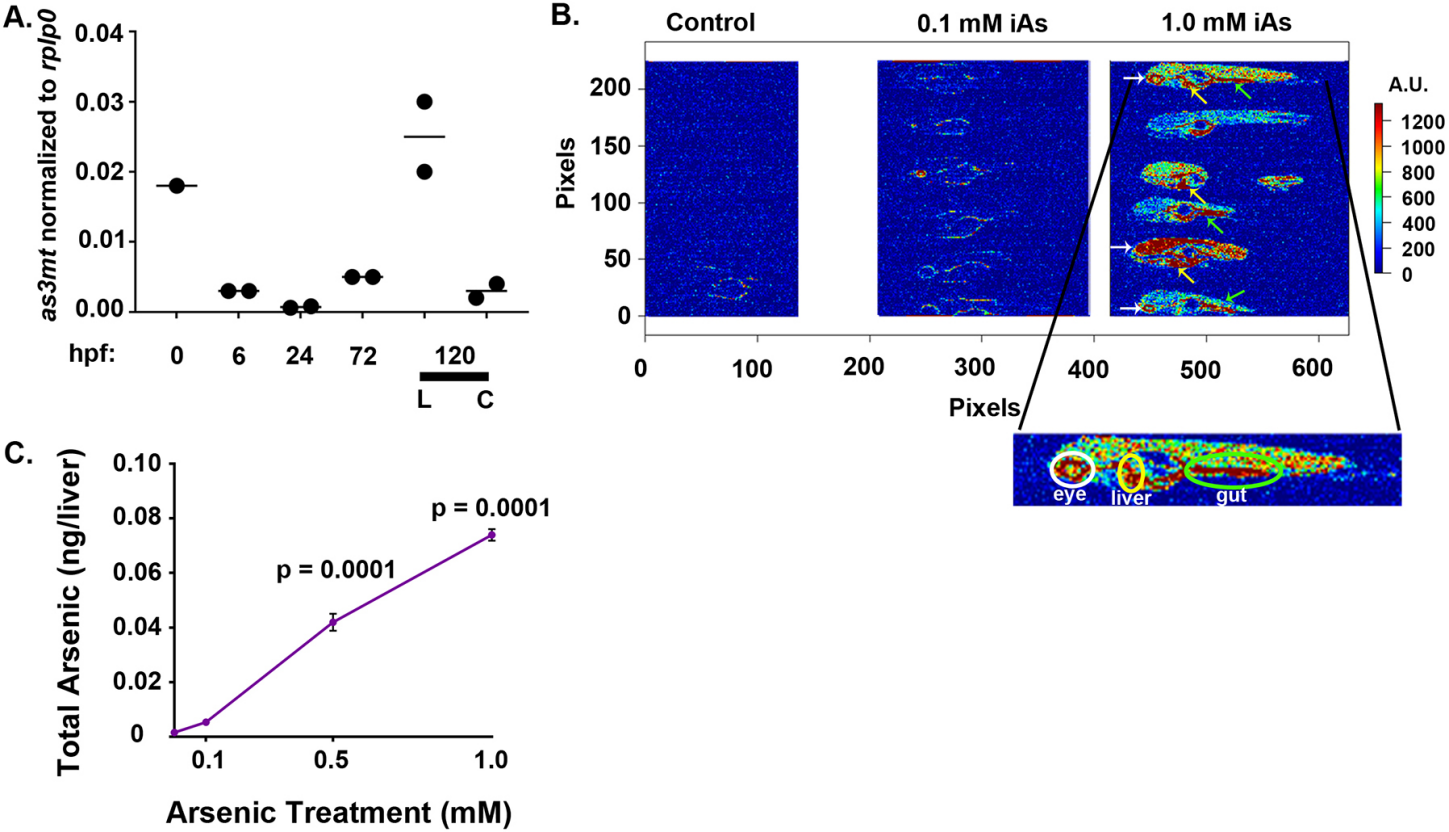
**Zebrafish are capable of arsenic metabolism and accumulate iAs in their tissues.** (A) Expression of the zebrafish *as3mt* transcript is dynamic during zebrafish development, as determined by qRT-PCR. *as3mt* is maternally provided. Expression is enriched in the liver at 120 hpf. Error bars correspond to mean±s.d. L, liver; C, carcass. (B) Representative images of LA-ICP-MS analysis of 10-μm sections of control and iAs-exposed larvae. Following exposure from 4 to 120 hpf, iAs accumulated in the eye (white arrows, white circle in enlarged image), liver (yellow arrows, yellow circle in enlarged image) and in the gut (green arrows, green circle in the enlarged image). Refer to Table S2 for operating parameters. (C) Quantification of total arsenic content in the livers of 5-dpf larvae by ICP-MS. Livers dissected from larvae exposed to 0, 0.1, 0.5 and 1.0 mM iAs from 4 to 120 hpf showed a dose-dependent increase in the total arsenic content per liver (*n*=3 clutches). Error bars correspond to mean±s.d. hpf, hours post-fertilization; LA-ICP-MS, laser-ablation–inductively-coupled-plasma–mass-spectroscopy; iAs, inorganic arsenic; ICP-MS, inductively-coupled-plasma–mass-spectroscopy.

Previous studies have demonstrated that zebrafish express aquaglyceroporins and the trivalent arsenic-specific methyltransferase (As3mt) (Hamdi et al., 2009, 2012), which are required for the uptake and metabolism of iAs, respectively. We asked whether zebrafish embryos expressed *as3mt* throughout development and determined whether, like in mammals (Thomas et al., 2004; Tseng, 2007), *as3mt* was enriched in the liver. Through both quantitative real-time PCR (qRT-PCR) analysis and mining transcriptomic data from Array Express (www.ebi.ac.uk/arrayexpress), it is clear that *as3mt* mRNA is maternally contributed and zygotically expressed (Fig. 2A, Fig. S2), and, at 5 dpf, expression was enriched by over 8-fold in the liver compared to the liver-less carcass (Fig. 2A).

We next asked whether iAs accumulated in zebrafish using laser-ablation–inductively-coupled-plasma–mass spectroscopy (LA-ICP-MS) (Hare et al., 2012) and inductively-coupled-plasma–mass spectroscopy (ICP-MS), highly sensitive microanalytical techniques used to detect elements in biological or geological samples. ICP-MS is best suited to provide a highly sensitive quantitative measurement of iAs levels and LA-ICP-MS is used for mapping the tissues where iAs accumulates (Sussulini et al., 2017). We found high concentrations of iAs in the skin, eye, gut and, most notably, liver in larvae exposed to 1 mM iAs from 4 to 120 hpf (Fig. 2B). ICP-MS performed on livers dissected from iAs-exposed and control larvae showed a highly consistent and dose-dependent increase in the amount of iAs with increasing exposure concentrations (Fig. 2C). These data show that zebrafish larvae express the enzyme required to metabolize iAs in the liver and that this is a site of iAs accumulation, suggesting this as a target tissue for iAs toxicity.

### iAs causes fatty liver

**Fig. 3.**
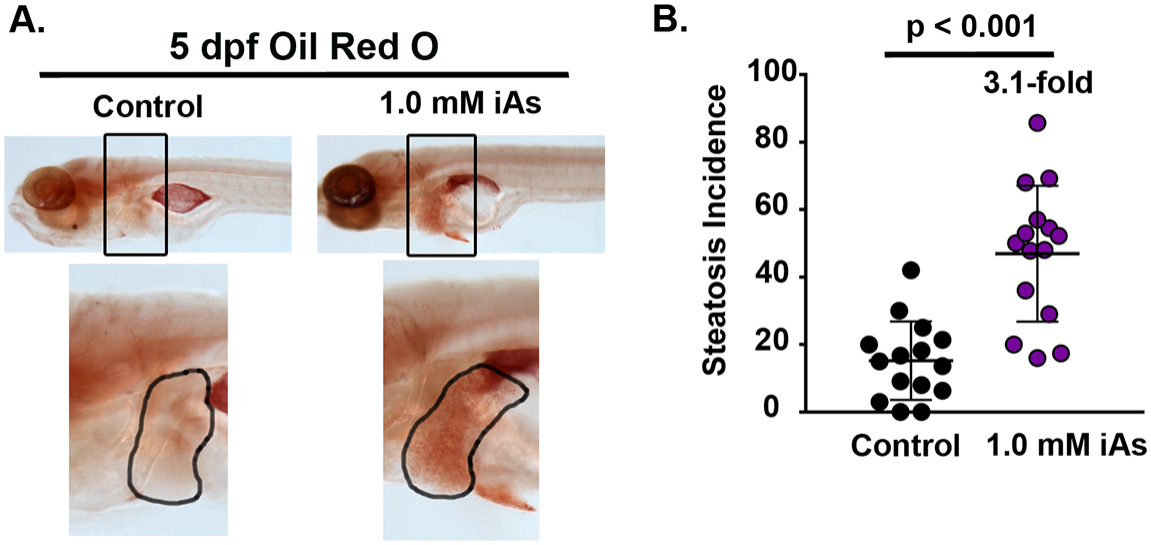
**Exposure to iAs causes steatosis in zebrafish larvae.** (A) Representative bright-field images of 5-dpf Oil Red O (ORO)-stained control and iAs-exposed (1.0 mM from 4 to120 hpf) larvae. The area around the liver (outlined in black) is enlarged. (B) The percent of larvae with steatosis analyzed by ORO staining of 15 clutches, with an average of 20 larvae per treatment per clutch. The total number of larvae analyzed in each clutch is listed in Table S1. Statistical significance was determined by unpaired, 2-tailed Student's *t*-test (*n*=15 clutches, *P*<0.001, Table S1). Error bars correspond to mean±s.d.

Chronic exposure to iAs is associated with altered liver function and liver disease (Das et al., 2012; Islam et al., 2011; Mazumder, 2005; Santra et al., 1999; Wang et al., 2014a,b). Previous studies in both mice and adult zebrafish showed that arsenic exposure can cause steatosis (Ditzel et al., 2016; Li et al., 2016; Tan et al., 2011) and can also sensitize mice to other factors that cause FLD, such as a high-fat diet (Tan et al., 2011). We tested the efficacy of iAs to directly cause FLD in zebrafish larvae treated with 1.0 mM iAs from 4 to 120 hpf using Oil Red O (ORO), which we have previously demonstrated is a straightforward means of assessing steatosis incidence across multiple clutches of larvae (Passeri et al., 2009; Vacaru et al., 2014) (Fig. 3A). The percent of control larvae with steatosis was an average of 15.2±3.0% (ranging from 0 to 42% across 15 clutches; Fig. 3B, Table S1). The incidence was significantly higher in the iAs-exposed larvae, with a mean of 46.9±5.2% (ranging from 16 to 85.7%, *n*=15 clutches, *P*<0.001) (Fig. 3B, Table S1). These data show that iAs is sufficient to cause FLD in this model, facilitating investigation of the cellular mechanisms of this phenotype.

### ER- and oxidative-stress responses are common pathways activated in response to either iAs or ethanol

**Fig. 4.**
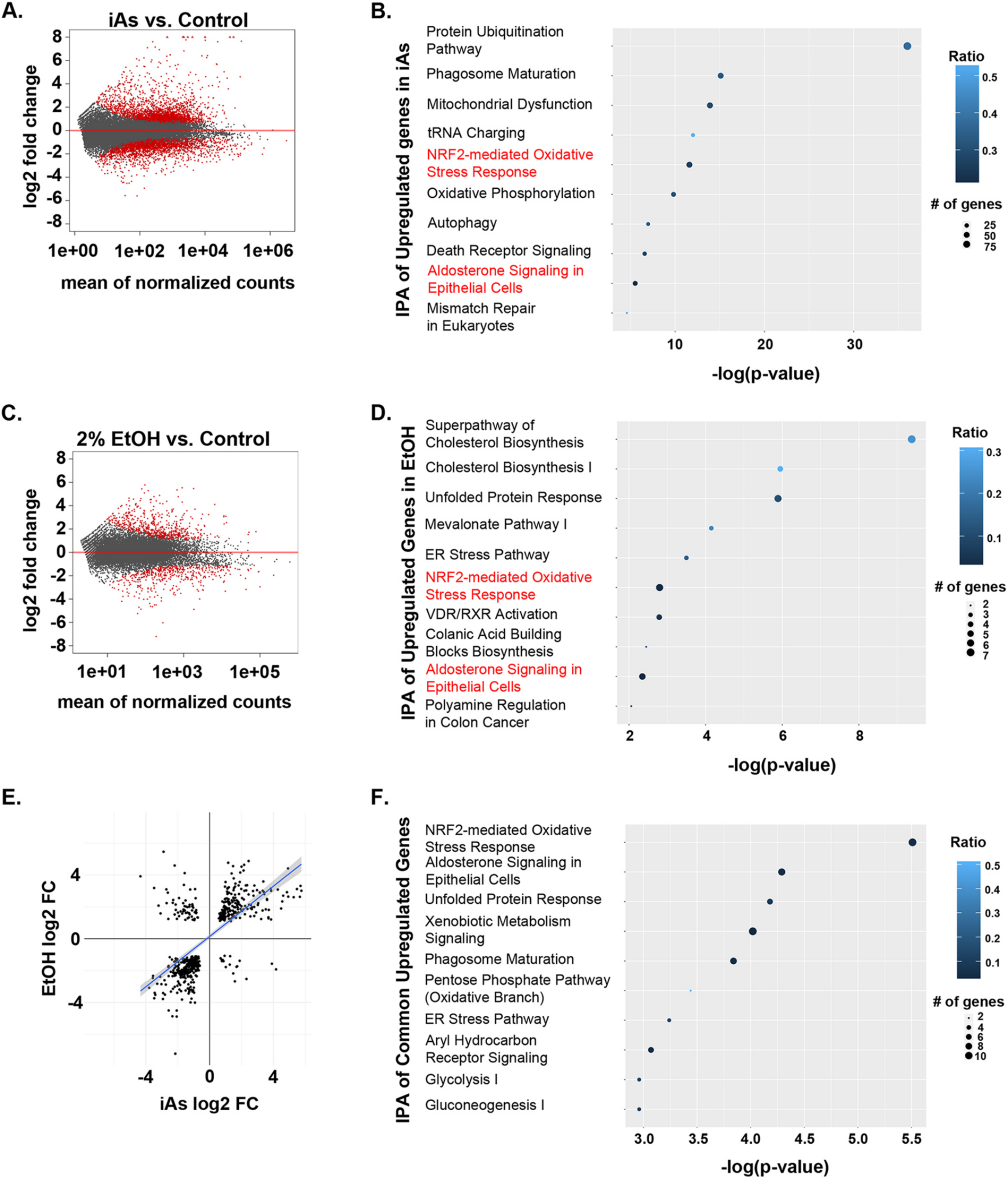
**Exposure to iAs induces expression of genes involved in metabolic processes and the UPR.** MA plot of normalized gene expression in livers from larvae exposed to 1.0 mM iAs from 4 to 120 hpf compared to unexposed siblings (A) and in livers from larvae exposed to 2% ethanol from 96 to 132 hpf compared to unexposed siblings (B). *P*-values and fold-changes for all significantly differentially expressed genes are included in Table S1. (B) IPA analysis of biological processes based on upregulated genes identified through RNAseq analysis. (C) MA plot of normalized gene expression in larvae exposed to 2% ethanol from 96-132 hpf compared to unexposed larvae. (D) IPA of biological processes based on upregulated genes identified through RNAseq analysis. (E) Plot of genes significantly upregulated in both the iAs and ethanol datasets (upper right quadrant), upregulated in the iAs dataset and downregulated in the ethanol dataset (lower right quadrant), downregulated in both the iAs and ethanol datasets (lower left quadrant), and upregulated in the ethanol dataset and downregulated in iAs dataset (upper left quadrant). (F) IPA of biological processes based on upregulated genes in both iAs and ethanol RNAseq datasets. iAs, inorganic arsenic; IPA, Ingenuity Pathway Analysis.

To identify cellular pathways activated in response to iAs and to gain insight into those processes that contribute to iAs-induced steatosis, we performed RNAseq analysis on livers dissected from 3 clutches of 5 dpf *Tg(fabp10a:nls-mCherry^mss4Tg^)* larvae exposed to 1 mM iAs from 4 to 120 hpf and from unexposed controls. In total, 5118 genes were differentially expressed between iAs-exposed and control siblings [adjusted *P*-value (padj)<0.05]; 2629 genes were upregulated (537 log2 fold change>2) and 2489 genes were downregulated (412 log2 fold change<−2) (Fig. 4A, Table 1, Table S3). In addition, some genes with read counts greater than or equal to 20 in one condition and less than or equal to 5 in the other were identified by our analysis. We designated these as ‘on-off’ genes, and found that 186 genes turned on and 214 genes turned off in the liver in response to iAs (Table 1). We identified pathways induced in the liver by iAs exposure using Ingenuity Pathway Analysis (IPA) of the upregulated genes in iAs samples. Metabolism (mitochondrial dysfunction and oxidative phosphorylation) and Nrf2-mediated oxidative stress pathways were highly enriched in the livers of iAs-exposed larvae (Fig. 4B), consistent with findings from other systems (Li et al., 2015; Sinha et al., 2013).

**Table 1.**
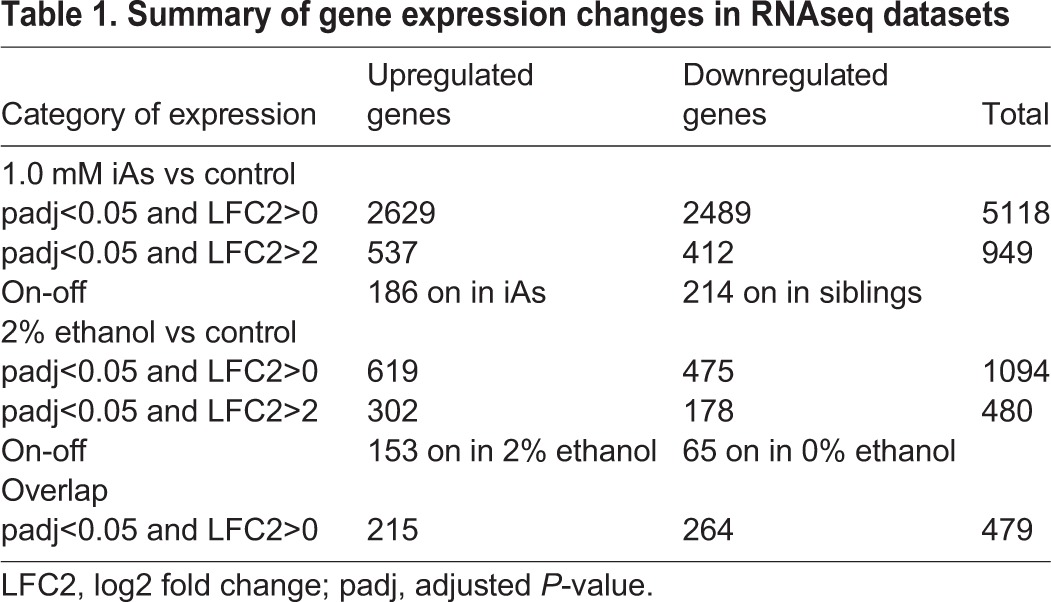
**Summary of gene expression changes in RNAseq datasets**

Reanalysis of an RNAseq dataset we previously generated from livers dissected from *Tg(fabp10a:nls-mCherry^mss4Tg^)* zebrafish exposed to 2% ethanol from 96 to 132 hpf (Howarth et al., 2014) was carried out to identify genes and pathways common to both iAs and ethanol treatment. We found significantly fewer differentially expressed genes (1094; padj<0.05), with 619 genes upregulated (302 log2 fold change>2) and 475 genes downregulated (178 log2 fold change<−2), with 153 genes on and 65 genes off in only ethanol-treated samples (Fig. 4C, Table 1, Table S3). IPA of all upregulated genes in the ethanol-treated group revealed that cholesterol biosynthesis (Superpathway of Cholesterol Biosynthesis, Cholesterol Biosynthesis I, Mevalonate Pathway), oxidative stress and UPR/ER-stress responses were among the most significantly enriched processes (Fig. 4D). This is consistent with our previous finding that UPR activation and FLD in response to alcohol was mediated by oxidative stress (Howarth et al., 2014; Tsedensodnom et al., 2013).

We found a high correlation between control samples in the two datasets, with correlation coefficients ranging from 0.87 to 1.0 (Fig. S3A). This allowed us to compare these two datasets to identify pathways that were commonly regulated. We identified 555 genes that were significantly differentially expressed in both the iAs and ethanol datasets (Fig. 4E, Table 1), of which 479 changed their expression in the same direction, with 215 genes upregulated and 264 genes downregulated in both datasets (Table 1, Table S3). IPA of the common upregulated genes revealed that oxidative stress, ER stress and UPR pathways were the most highly represented (Fig. 4F). Oxidative stress generated during iAs metabolism is a proposed mechanism of arsenic toxicity (Huang et al., 2004; Santra et al., 2000a, 2007). Decades of research in mammalian systems (Lu and Cederbaum, 2008; Magdaleno et al., 2017) and our work in zebrafish (Tsedensodnom et al., 2013) have shown that oxidative stress is also a primary cellular mechanism of ALD. Interestingly, 3 of the commonly regulated pathways (Pentose Phosphate Pathway, Glycolysis, and Gluconeogenesis) were previously found to be induced in transcriptome or metabolomics analysis of livers from adult zebrafish acutely exposed to iAs (Li et al., 2016; Xu et al., 2013).

**Fig. 5.**
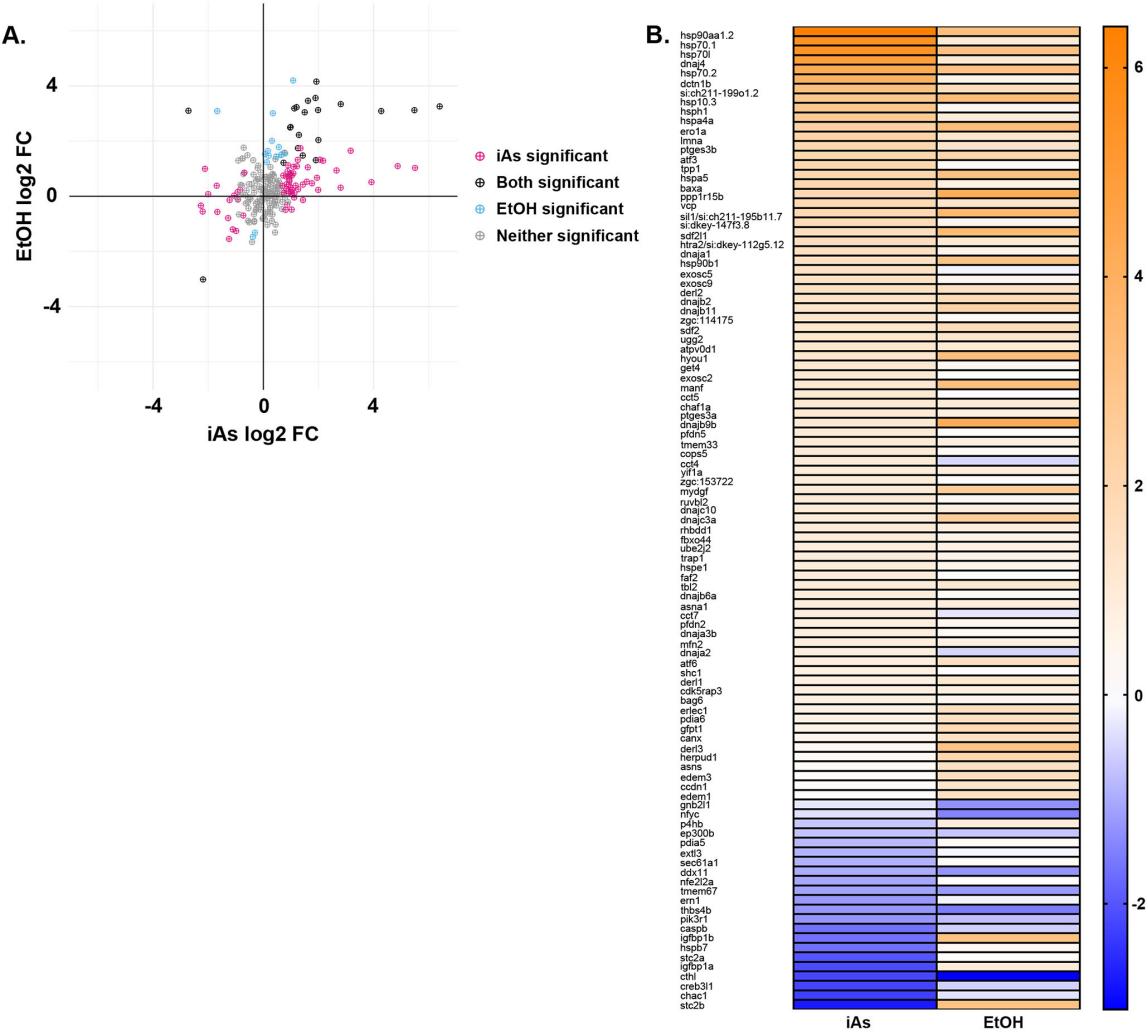
**Arsenic and ethanol regulate common cellular pathways.** (A) Plot of UPR-associated gene expressions in iAs and ethanol RNAseq datasets. (B) Heat map of the expression of 103 significantly differentially regulated UPR genes. Refer to Table S4 for *P*-values and fold-changes. iAs, inorganic arsenic.

Different stressors induce distinct UPRs (Vacaru et al., 2014) and each branch of the UPR modulates protein folding in a unique way. We analyzed the iAs and ethanol RNAseq datasets to determine whether these toxicants also activated distinct UPRs. We generated a list of 254 genes that are targets of the UPR or function in the UPR based on annotations in AmiGo and NCBI. Of these UPR-associated genes, the majority (227 genes) were expressed in both the iAs and ethanol RNAseq datasets and nearly half of these (103 genes) were significantly differentially expressed in one or both datasets (Fig. 5A, Table S4), with 89 differentially expressed in the iAs dataset and 37 differentially expressed in the ethanol dataset. Interestingly, the pattern of UPR gene expression was largely distinct in these two models, as only 10% of all UPR-associated genes and 20% of genes differentially expressed in one of the datasets showed a shared expression pattern in both datasets (Fig. 5A,B). The UPR-associated genes affected by both stressors include the *bona fide* UPR genes *atf3*, *hspa5*, *hyou1* and *dnajc3*, as well as heat-shock-related genes (Fig. 5B, Table S4). Additionally, 67 of the UPR-associated genes were only changed in the iAs dataset and 15 genes were only changed in the ethanol dataset (Fig. 5A, Table S4). Because most of the UPR-associated transcripts were detected in both datasets at similar levels based on FPKM (fragments per kilobase of transcript per million mapped reads) values (Fig. S3B), we concluded that the unique patterns of UPR gene expression in these two datasets reflect distinct types of UPRs [i.e. UPR subclasses (Vacaru et al., 2014)]. We used qRT-PCR to confirm that the UPR genes we previously showed to be associated with FLD [*xbp1*, *xbp1s*, *bip*, *chop*, *dnajc3*, *edem1*, *atf4* and *atf6* (Vacaru et al., 2014)] were upregulated in the liver of iAs-exposed larvae, and found that all except *xbp1* and *xbp1s* were significantly induced (*n*=9 clutches, *P*<0.01) (Fig. S4A). We functionally assessed the relevance of ER stress to iAs-induced toxicity by co-exposing larvae to iAs and tunicamycin, a well-characterized and widely used ER stressor that we have shown causes FLD in zebrafish (Cinaroglu et al., 2011; Howarth et al., 2013; Vacaru et al., 2014), and found them to be synergistically lethal (Fig. S4B), suggesting that they act through shared mechanisms to cause lethality.

Together, these data indicate that (1) similar pathways are deregulated by iAs and ethanol, or ethanol alone, including oxidative stress and the UPR, (2) both iAs and ethanol induce the UPR, that (3) a subset of UPR genes are targeted by both iAs and ethanol, and that (4) each of these toxicants induces a unique UPR signature, extending our previous findings of distinct UPR subclasses in response to other ER stressors (Vacaru et al., 2014). We speculate that the subset of UPR genes deregulated in response to both stressors reflects some commonality in their mechanism of UPR induction.

### Ethanol potentiates iAs toxicity, ER stress and fatty liver

**Fig. 6.**
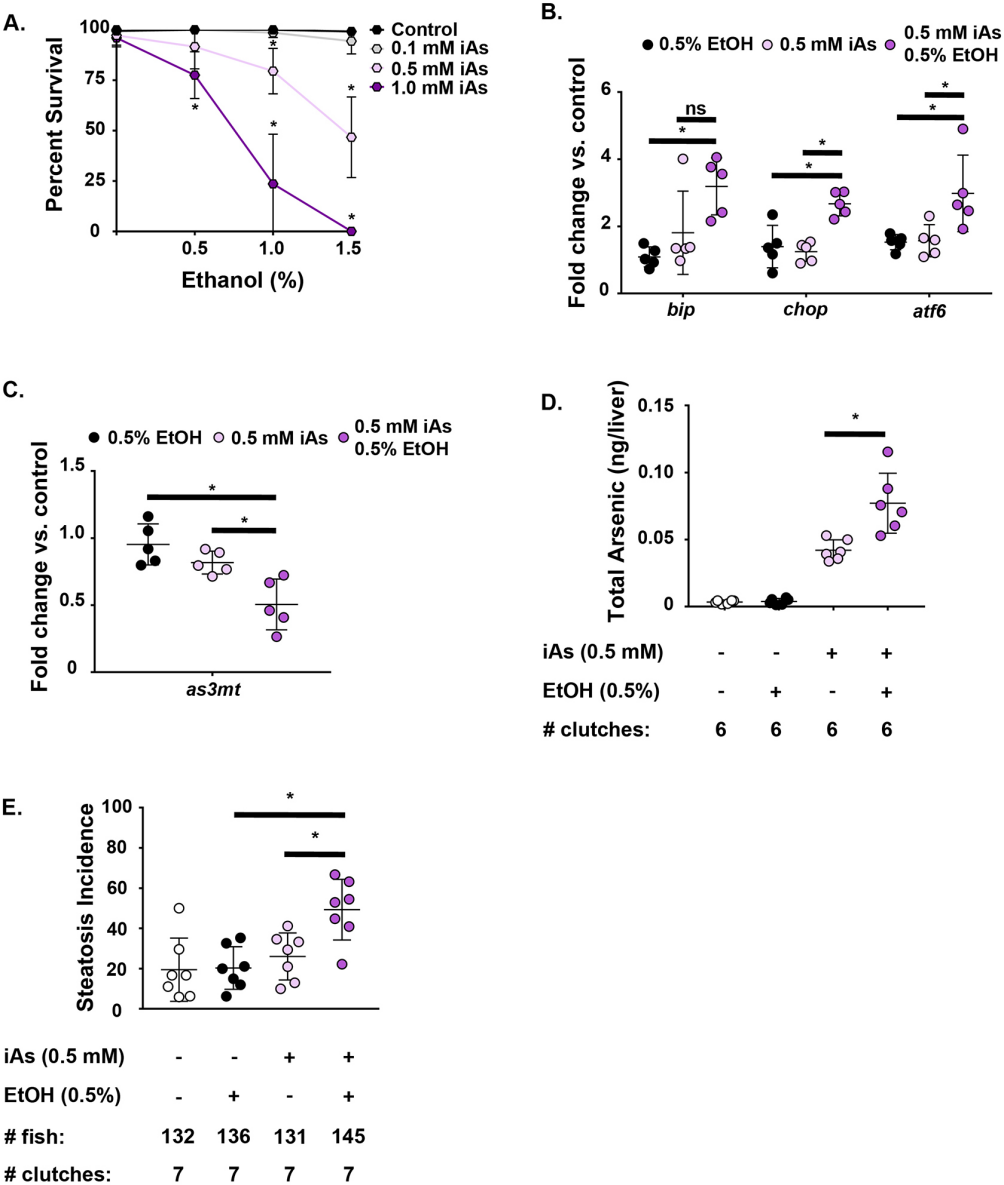
**Arsenic potentiates alcohol-induced liver disease in zebrafish larvae.** (A) Survival of zebrafish larvae at 120 hpf. Zebrafish larvae were exposed to a range of concentrations of iAs and/or ethanol. Survival was assessed at 120 hpf. Statistical significance was determined by unpaired, 2-tailed Student's *t*-test, correcting for multiple comparisons (*n*=3 clutches, *P*<0.001). Data are presented as mean±s.d. (B) qRT-PCR data from liver cDNA. Data are presented as fold-change vs control. Statistical significance was determined by one-way ANOVA (*n*=5 clutches, *bip*: 0.5% ethanol vs 0.5 mM iAs+0.5% ethanol *P=0.0072, 0.5 mM iAs vs 0.5 mM iAs+0.5% ethanol *P*=0.078; *chop*: 0.5% ethanol vs 0.5 mM iAs+0.5% ethanol *P=0.0021, 0.5 mM iAs vs 0.5 mM iAs+0.5% ethanol *P=0.0008; *atf6*: 0.5% ethanol vs 0.5 mM iAs+0.5% ethanol *P=0.0205, 0.5 mM iAs vs 0.5 mM iAs+0.5% ethanol *P=0.0241). (C) qRT-PCR data from liver cDNA. Data are presented as fold-change vs control. Statistical significance was determined by one-way ANOVA (*n*=5 clutches, 0.5% ethanol vs 0.5 mM iAs+0.5% ethanol *P=0.0013, 0.5 mM iAs vs 0.5 mM iAs+0.5% ethanol *P=0.0158). (D) Quantification of total arsenic content in the livers of 5-dpf larvae by liquid ICP-MS. Statistical significance was determined by one-way ANOVA (*n*=6 clutches, 0.5 mM iAs vs 0.5 mM iAs+0.5% ethanol *P=0.0003). (E) Steatosis incidence in 120-hpf larvae exposed to 0.5 mM iAs, 0.5% ethanol and 0.5 mM iAs+0.5% ethanol. Statistical significance was determined by one-way ANOVA (*n*=7 clutches, 0.5% ethanol vs 0.5 mM iAs+0.5% ethanol *P=0.0025, 0.5 mM iAs vs 0.5 mM iAs+0.5% ethanol *P=0.0172). Error bars in all panels correspond to mean±s.d.

Our finding that there are shared pathways targeted by both ethanol and iAs suggests that combined exposure may interact to cause liver disease. To test this, embryos were treated with a range of concentrations of iAs (0.1 mM, 0.5 mM, 1.0 mM) starting at 4 hpf and subsequently exposed at 96 hpf to a range of ethanol concentrations (0.5%, 1.0%, 1.5%) which we have previously shown are below and above the threshold for causing lethality and FLD (Tsedensodnom et al., 2013). We found that iAs significantly reduced survival of larvae exposed to ethanol concentrations that were otherwise well tolerated on their own (*n*=3 clutches, *P*<0.001) (Fig. 6A), showing that they synergize to cause lethality and suggesting that this results from their convergence on the same pathways.

To investigate the nature of this interaction, we reduced exposure concentrations to ensure survival of co-exposed larvae to allow further analysis. qPCR analysis of samples from larvae exposed to 0.5% ethanol or 0.5 mM iAs alone or in combination revealed that co-exposure enhanced expression of *bip*, *chop* and *atf6*, compared to that in samples treated with either toxicant alone (Fig. 6B). This demonstrates that exposure to a dose of iAs that does not cause any morphological or gene expression changes can predispose zebrafish larvae to a dramatic response to a low dose of ethanol. This is important as it suggests that co-exposure to sub-toxic concentrations of these two toxicants can cause disease.

We next tested our hypothesis that a potential mechanism by which iAs and ethanol could interact would be via altered expression of the enzymes responsible for iAs metabolism and clearance. We found that *as3mt* expression was significantly reduced in fish treated with iAs and ethanol compared to those exposed to either toxicant alone (*n*=5 clutches, 0.5% ethanol vs 0.5 mM iAs+0.5% ethanol *P*=0.0013, 0.5 mM iAs vs 0.5 mM iAs+0.5% ethanol *P*=0.0158) (Fig. 6C). Because reduced expression of *as3mt* can impair clearance of iAs from the liver, we hypothesized that ethanol exposure would increase the iAs levels in the liver. ICP-MS analysis showed that iAs levels were significantly higher in the livers of larvae exposed to 0.5 mM iAs plus 0.5% ethanol than in larvae exposed to 0.5 mM iAs alone (*n*=6 clutches, *P*=0.0003) (Fig. 6D). This demonstrates that the clearance of arsenic from the liver is hindered upon co-exposure to these two toxicants.

**Fig. 7.**
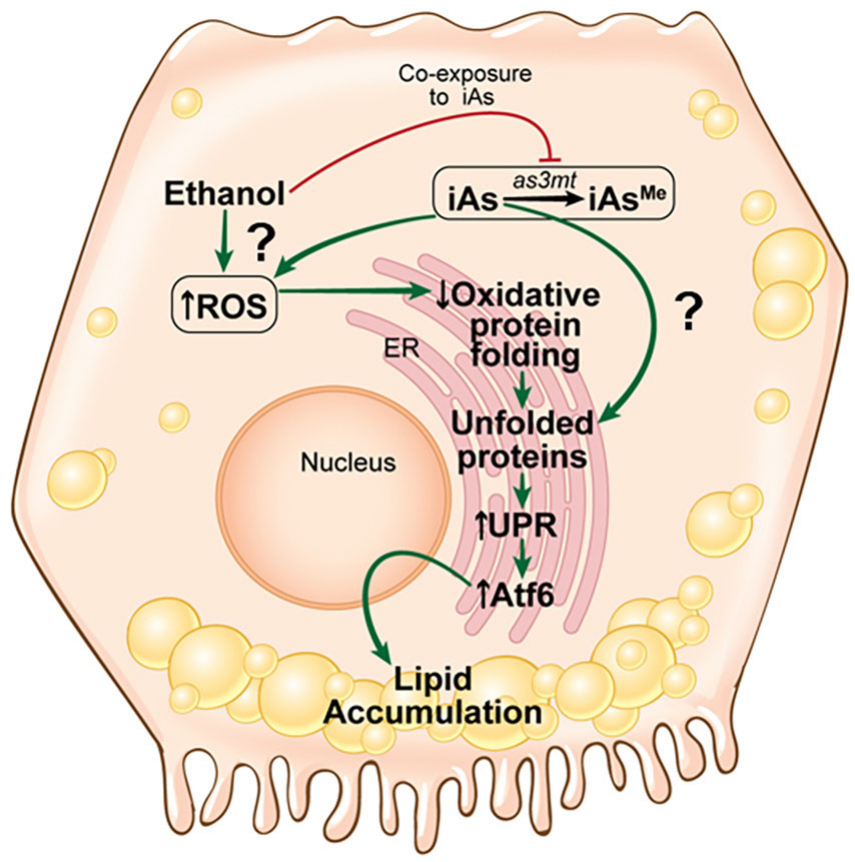
**Model of the mechanism by which iAs and ethanol interact to cause ER stress.** iAs and ethanol interact to increase the concentration of iAs in the liver, aggravate ER stress and enhance FLD. This figure was drawn by J. Gregory (Mount Sinai Health System).

We next hypothesized that accumulation of iAs can increase the toxic sequelae of iAs exposure, including exacerbating ER stress and potentially increasing FLD incidence. To test this, we exposed zebrafish to each toxicant alone or in combination at doses which, when given independently, do not cause steatosis. Zebrafish embryos were treated with 0.5 mM iAs at 4 hpf, followed by addition of either 0 or 0.5% ethanol at 96 hpf and collected at 120 hpf for ORO staining. We found that the incidence of steatosis was significantly higher in larvae exposed to both toxicants compared to those exposed to either agent alone (*n*=7 clutches, 0.5% ethanol vs 0.5 mM iAs+0.5% ethanol *P*=0.0025; 0.5 mM iAs vs 0.5 mM iAs+0.5% ethanol *P*=0.0172) (Fig. 6E, Table S5). To determine whether we could detect an interaction between iAs and ethanol in the development of steatosis, we modeled the risk difference between the exposure conditions. The combined incidence of steatosis across all 7 clutches was determined by dividing the number of larvae with steatosis in each group by the total population of that group. This yielded a background incidence of steatosis of 20.45% (27/132) in control larvae, 19.85% (27/136) in larvae exposed to 0.5% ethanol alone, 25.19% (33/131) in larvae exposed to 0.5 mM iAs alone, and 48.97% (71/145) in larvae co-exposed to 0.5 mM iAs+0.5% ethanol (Fig. 6E, Table S5). Controlling for the fact that some of the larvae were from the same clutch, neither exposure to 0.5% ethanol nor 0.5 mM iAs alone significantly increased the risk of developing steatosis relative to control (0.5% ethanol *P*=0.98, 0.5 mM iAs *P*=0.36). However, co-exposure to 0.5 mM iAs+0.5% ethanol resulted in an increased risk of developing steatosis at an alpha of 0.1, commonly used to assess the risk of interaction in epidemiological studies (*P*=0.08). These data support the conclusion that iAs and ethanol interact to increase the concentration of iAs in the liver, aggravate ER stress and enhance FLD (Fig. 7).

## DISCUSSION

Genetic and lifestyle factors are assumed to be primary causes of FLD; however, environmental toxicants can serve important disease-modifying roles by targeting cellular pathways or processes that render them more susceptible to other stimuli. Here, we show that high doses of iAs causes FLD and can modify the risk of ALD. Importantly, the interaction between iAs and ethanol is observed at doses of these toxicants which alone do not cause any detectable phenotypic or cellular changes. This suggests that exposure to a sub-toxic level of iAs could serve as a risk factor for more common causes of liver disease, such as ALD. Additionally, our unbiased approach to identify pathways that serve as a nexus for these toxicants revealed oxidative and ER stress as points of convergence. We hypothesize that these cellular stress responses are the mechanisms for the increased toxicity we observe in the presence of iAs and ethanol. Finally, we present mechanistic data demonstrating that ethanol acts to decrease *as3mt* expression, thereby reducing iAs metabolism, leading to sustained, high concentrations of iAs in the liver and potentiating its toxic impact.

Oxidative stress is a major cause of hepatic injury caused by alcohol because ROS are generated from alcohol metabolism by the cytochrome P450 system (CYP2E1) (Cederbaum, 2010; Lu and Cederbaum, 2008). Previous work from our lab (Tsedensodnom et al., 2013) and others (Ashraf and Sheikh, 2015; Cederbaum, 2010) has shown that ROS generated during ethanol metabolism leads to ER stress. Protein folding in the ER is dependent on a delicate redox balance in order to form the disulfide bonds that are required for proper protein structure. By altering the redox balance in the ER, oxidative protein folding is impaired (Hudson et al., 2015), leading to UPR induction to mitigate the accumulation of unfolded proteins (Malhotra and Kaufman, 2007). An important insight into arsenic-mediated pathology is the finding that iAs and its metabolic derivatives act as strong inhibitors of oxidative protein folding (Jacobson et al., 2012; O'Connell et al., 2014; Sapra et al., 2015) and bind to unfolded proteins (Ramadan et al., 2009), and that trivalent iAs species directly prevent the proper folding of nascent peptides due to strong affinity for thiol groups on cysteine residues (Ramadan et al., 2009; Watanabe and Hirano, 2013). Our working model (Fig. 7) proposes that iAs exposure increases the unfolded protein load in the ER by directly acting as a reducing agent, and indirectly by disrupting the redox balance in the ER through ROS generated during iAs metabolism by As3mt. Given that our RNAseq analysis found that a CYP450 enzyme that metabolizes ethanol in zebrafish (*cyp2y3*; Tsedensodnom et al., 2013) is downregulated in response to iAs, it is unlikely that iAs treatment potentiates ethanol metabolism; if anything, it may reduce it.

Our model suggests that iAs induces FLD by activating a pathological or ‘stressed’ UPR subclass (Howarth et al., 2014; Tsedensodnom et al., 2013; Vacaru et al., 2014; Wang and Kaufman, 2016). We speculate that some aspects of this stressed UPR are shared with the response to ethanol. We demonstrated that Atf6 is necessary and sufficient for fatty liver in zebrafish (Cinaroglu et al., 2011; Howarth et al., 2014) and, because *atf6* is one of the few genes that is targeted by both iAs and ethanol, we speculate that FLD caused by iAs is mediated by Atf6 activation. However, it is also possible that the unique aspects of the UPR which are induced by iAs and by ethanol could create unique proteostatic environments that are only minimally overlapping, as found in mammalian cells in culture (Shoulders et al., 2013).

Other work has suggested that iAs impacts mitochondrial function (Luz et al., 2016; Santra et al., 2007) and our RNAseq analysis also highlighted mitochondrial dysfunction as a response to iAs exposure. It is possible that mitochondrial damage in hepatocytes could reduce lipid oxidation and cause lipid accumulation. Another interesting possibility is that communication between the mitochondria and ER could impair the function of both of these organelles if one is damaged. We found that *ero1a*, a component of mitochondrial-associated ER membranes (MAMs) and a regulator of oxidative protein folding and ER redox homeostasis (Anelli et al., 2011; Benham et al., 2013; Seervi et al., 2013), is among the genes that are significantly upregulated in both iAs and ethanol datasets. MAMs are key signaling centers that act at the interface between mitochondria and the ER, and are linked by their key functions in redox homeostasis and calcium storage (Carreras-Sureda et al., 2017). Thus, it is possible that defects in the function of multiple organelles contribute both to the ER stress and FLD phenotypes caused by iAs exposure.

Other mechanisms proposed to mediate iAs toxicity – namely changes in DNA methylation (Huang et al., 2004; Hughes, 2002; Kile et al., 2012; Mauro et al., 2016) – were not identified by our studies in zebrafish (not shown). In fact, there is nearly no overlap in the gene expression changes induced by iAs and by mutants that have a defect in DNA methylation (Chernyavskaya et al., 2017; Jacob et al., 2015), and our preliminary studies found neither an impact on global DNA methylation nor synergy with mutants that have DNA methylation loss (not shown). Thus, we conclude that oxidative and ER stress are the major mechanisms of iAs hepatotoxicity.

By comparing the iAs and ethanol RNAseq datasets, we found several additional enriched pathways that may contribute to the development of FLD. Both glycolysis and gluconeogenesis pathways were enriched in both datasets, and these pathways have previously been demonstrated to be induced by iAs in the liver of guinea pigs and adult zebrafish (Li et al., 2016; Reichl et al., 1988). We speculate that these pathways are important for maintaining the energetic balance required for efficient protein folding and hepatic metabolism, and that the accumulation of lipids in hepatocytes of toxin-stressed cells may serve as an adaptive function to store energy.

Although our RNAseq analysis and other data provide evidence that iAs and ethanol are converging on the same cellular pathways, it is important to note that there are differences in the gene expression profiles in response to the two toxicants. It is therefore possible that the cumulative effects of independent cellular responses are converging to yield the phenotypes analyzed in this study (i.e. FLD and death). The same zebrafish line, *Tg(fabp10a:nls-mCherry^mss4Tg^)*, was used to generate both RNAseq datasets, and consistency across sample maintenance (i.e. light:dark cycle, culture conditions and time of collection) were maintained to provide a basis for comparison. Moreover, qPCR analysis of additional clutches that were treated in parallel confirmed that the gene expression changes detected by RNAseq were reproducible.

An important finding from our work is that co-exposure to iAs and ethanol reduces the expression of *as3mt*, which catalyzes the methylation of iAs and results in an increase in the total accumulation of arsenic in the liver. Polymorphisms in the human *AS3MT* gene are predictive of arsenic metabolism across multiple study populations (Engstrom et al., 2011, 2013). Indeed, reduced capacity for arsenic methylation has previously been shown to correlate with adverse outcomes following arsenic exposure (Chen et al., 2013; Das et al., 2016). Human genome-wide association studies for mutations promoting arsenic susceptibility identified an *AS3MT* haplotype that leads to reduced expression of *AS3MT* and increased toxicity (Das et al., 2016). Recently it was shown that C57BL/6J mice are more susceptible to arsenic-induced oxidative liver injury than 129X1/SvJ mice, and that this difference is related to their reduced capacity for arsenic methylation (Wu et al., 2017). In addition, one study found that alcohol use may decrease methylation of iAs (Hopenhayn-Rich et al., 1996; Tseng, 2009). Future research will seek to determine the mechanisms by which ethanol reduces expression of *as3mt*.

Although zebrafish are at the forefront of toxicology research (Bambino and Chu, 2017; Garcia et al., 2016), there are some limitations in the extension of this study to understanding the effects of arsenic and alcohol on liver disease in humans. As with other experimental models, translation of the results to the effects of human exposures are complicated by differences in arsenic metabolism (Vahter, 1999) and the doses used under laboratory conditions (States et al., 2011). The concentration of iAs used here surpasses the levels that are associated with disease in humans, suggesting that, in this system where animals are immersed in iAs, low levels are not acutely toxic, but instead may have an impact over longer-term exposures. We have used exposures in the low parts per million (ppm) range, whereas humans are typically exposed to arsenic concentrations in the parts per billion (ppb) range, with a WHO standard of 10 ppb. However, samples of drinking water from Argentina, Bangladesh, China, Taiwan and the United States have all been found to contain iAs at concentrations greater than 2 mg l^−1^ (2 ppm) (Naujokas et al., 2013), indicating the possibility of exposure at levels higher than previously presumed. We examined the effect of arsenic exposure beginning just prior to gastrulation to mimic the effects of early-life exposure; however, this does not completely recapitulate the predominant route of human exposure, as the zebrafish do not begin to swallow water until 3 dpf.

Collectively, these data demonstrate that combined exposure to iAs and ethanol led to a reduced survival of zebrafish larvae, enhanced induction of the UPR and an increase in the risk of fatty liver disease. Given the high conservation of common disease-related genes and pathways between zebrafish and humans (Goessling and Sadler, 2015), we hypothesize that the mechanism of iAs-induced FLD and the cellular pathways targeted by both toxicants will also be conserved. With the increasing global incidence of liver disease, the risk of liver damage caused by iAs exposure may be more significant than previously presumed.

## MATERIALS AND METHODS

### Zebrafish maintenance and treatment of embryos

Procedures were performed in accordance with the Icahn School of Medicine at Mount Sinai Institutional Animal Care and Use Committee (IACUC). Adult wild-type (WT; AB, Tab14 and TAB5) and *Tg(fabp10a:nls-mCherry^mss4Tg^)* (Mudbhary et al., 2014) fish were maintained on a 14:10 light:dark cycle at 28°C. Fertilized embryos from natural spawning of group matings were collected and cultured in embryo water (0.6 g/l Crystal Sea Marinemix; Marine Enterprises International, Baltimore, MD) containing Methylene Blue at 28°C. Embryos were treated with sodium (meta)arsenite (Sigma, S7400) beginning at 4 h post fertilization (hpf). Sodium (meta)arsenite and/or ethanol were diluted from stock solutions in 10 ml of embryo water. After addition of exposure medium, 35-mm dishes were sealed with Parafilm and returned to the incubator. Medium was not replaced during the exposure period unless otherwise noted. For co-exposures, sodium (meta)arsenite was removed and replaced with embryo water containing sodium (meta)arsenite and tunicamycin at 72 hpf or sodium (meta)arsenite and ethanol at 96 hpf at the indicated concentrations. Images of anesthetized larvae were taken by mounting in 3% methyl cellulose using a Nikon SMZ1500 stereomicroscope.

### Gene expression analysis by qRT-PCR

Livers were microdissected in from anesthetized 5 dpf zebrafish larvae that were immobilized in 3% methyl cellulose using 30 gauge needles and collected in 20 µl RLT Buffer (Qiagen), and RNA was isolated by extraction in TRIzol Reagent (Life Technologies) as described (Passeri et al., 2009). RNA (250 ng) was reverse transcribed using the SuperScript cDNA synthesis kit (Quanta) as per the manufacturer's instructions. qRT-PCR was performed using PerfeCTa SYBR Green Fast Mix (Quanta). Samples were run in triplicate on the Roche LightCycler 480 (Roche), with at least three independent samples analyzed for each experiment. Target gene expression was normalized to *ribosomal protein large P0* (*rplp0*) using the comparative threshold cycle (ΔΔCt) method. Expression in treated animals was normalized to untreated controls from the same clutch. Primer sequences are listed in Table S6.

### mRNA sequencing (RNAseq)

Total RNA was extracted from livers dissected from 5 dpf *Tg(fabp10a:nls-mCherry^mss4Tg^)* zebrafish larvae using the Zymo Quick-RNA Micro Kit (R1050, Zymo Research) as per the manufacturer's instructions. RNA quality was determined by Agilent 2100 BioAnalyzer. RNAseq libraries were prepared according to Illumina TruSeq RNA sample preparation version 2 protocol, and library quality was analyzed on the Agilent 2100 BioAnalyzer. cDNA libraries were sequenced on the Illumina Hiseq 2500 platform to obtain 100-base-pair paired-end reads. Sequencing quality was assessed by using FASTQC (https://www.bioinformatics.babraham.ac.uk/projects/fastqc/), and reads were quality trimmed using Trimmomatic (Bolger et al., 2014) to remove low Q scores, adapter contamination and systematic sequencing errors. Reads were aligned to the *Danio rerio* GRCz10 reference genome assembly with Tophat 2.0.9 (Trapnell et al., 2009). To estimate gene expression, read counts were calculated by HTSeq with ensemble annotation (Aken et al., 2016; Anders et al., 2015; Trapnell et al., 2009). Normalization and test of differential expression using a generalized linear model were implemented in DESeq2 (Gentleman et al., 2004; Love et al., 2014). Adjusted *P*-value (FDR) <0.05 was treated as significantly different expression. We established a method to identify ‘on-off’ genes as those that were expressed in controls but not in the experimental condition, or vice versa. These were designated as genes with read counts greater than or equal to 20 in one condition and less than or equal to 5 in the other. RNAseq datasets were submitted to Gene Expression Omnibus (GEO) with the access number GSE104953. RNAseq from livers of *Tg(fabp10a:nls-mCherry^mss4Tg^)* larvae exposed to 2% ethanol from 96 to 132 hpf was previously described (Howarth et al., 2014; GSE56498), as were the unexposed 5 dpf controls (GSE52605; Mudbhary et al., 2014). The data was realigned and reanalyzed for this study.

### Qualitative and quantitative assessment of iAs content in zebrafish larvae

Total iAs analysis was carried out by ICP-MS on pools of 20 dissected livers from 5-dpf larvae in 20 μl deionized water followed by 0.5 ml of concentrated double-distilled nitric acid. After an overnight digestion at room temperature, samples were diluted to 5 ml and analyzed using ICP-MS (Agilent 8800-QQQ, Wilmington, DE). iAs concentration was measured in MS/MS mode using oxygen as the cell gas and tellurium as the internal standard. Quality control (QC) and quality assurance procedures included analyses of procedural blanks and QC standards. Lab recovery rates for QC standards with this method were 90-110% and <5% precision (given as %RSD). The limit of detection for arsenic was 0.02 ng ml^−1^.

For tissue mapping by LA-ICP-MS, larvae were washed with embryo water and fixed in 4% paraformaldehyde (PFA) in phosphate buffered saline (PBS) overnight at 4°C. They were transferred to 30% sucrose in PBS and embedded in a 2:1 mixture OCT (Tissue Tek):30% sucrose. Cryosections of 10 μm were cut on a Leica CM3050S cryostat, and were thawed and warmed to room temperature prior to analysis.

A New Wave Research NWR-193 (ESI, OR, USA) laser ablation unit equipped with a 193 nm ArF excimer laser was connected to an Agilent Technologies 8800 triple-quad ICP-MS (Agilent Technologies, CA, USA). Helium was used as the carrier gas from the laser ablation cell and mixed with argon via Y-piece before introduction to the ICP-MS. The system was tuned daily using NIST SRM 612 (trace elements in glass) to monitor sensitivity (maximum analyte ion counts), oxide formation (232Th16O+/232Th+, <0.3%) and fractionation (232Th+/238U+, 100±5%). The laser was scanned across the tissue sections at 20 μm spot size, 40 μm/s scan speed, 40 Hz repetition rate and approximately 0.2 J cm^2^ power. ICP-MS integration times for analytes were adjusted so that total scan time was 0.5 s, maintaining the dimensions of the tissue in data analysis. LA-ICP-MS operating parameters are given in Table S2.

### Lipid staining

Larvae were fixed in 4% PFA overnight at 4°C and stained with Oil Red O as previously described (Passeri et al., 2009; Vacaru et al., 2014). Samples were blinded before scoring as positive (presence of 3 or more lipid droplets per liver) or negative for steatosis. The average steatosis incidence was calculated for at least 3 clutches per condition and, on average, 15-20 larvae per condition per experiment were scored (Tables S3 and S6).

### Statistical analysis

Data were analyzed using GraphPad Prism 7 software, and interaction between iAs and ethanol in steatosis was modeled in SAS version 9.4 (SAS Institute Inc., Cary, NC). Data are expressed as means±s.d. Differences between experimental groups were analyzed by unpaired Student’s *t*-test (with correction for multiple comparisons where applicable), or by one-way analysis of variance (ANOVA) followed by Dunnet's or Tukey *post hoc* correction when more than two groups were compared. *P*-values <0.05 were considered statistically significant unless otherwise noted. Differentially expressed genes from RNAseq data were analyzed using a package DESeq2 in Bioconductor (Gentleman et al., 2004; Love, 2014), which embedded a generalized linear model for test of differential expression. Adjusted *P*-value (FDR) <0.05 was treated as significantly differential expression.

## Supplementary Material

Supplementary information

## Supplementary Material

First Person interview
