# Supplementary material for: Inorganic arsenic causes fatty liver and interacts with ethanol to cause alcoholic liver disease in zebrafish
Source: Dis Model Mech. 2018 Feb 1;11(2):dmm031575. doi: 10.1242/dmm.031575 (PMC5894941; doi:10.1242/dmm.031575)
Supplement: Supplementary information [file dmm-11-031575-s1.pdf]

Table S1. Number of larvae analyzed per experiment for Figures 1 through 3.

Click here to Download Table S1

Table S2. LA-ICP-MS operating parameters.

NWR-193 Laser Conditions		Agilent 8800 ICP-MS Conditions	
**Wavelength (nm)**	193	RF power (W)	1350
**Helium carrier flow (L min^{-1})**	0.8	Argon carrier flow (L min^{-1})	0.6
**Fluence (J cm^{-2})**	0.2	Plasma gas flow (L min^{-1})	15
**Repetition rate (Hz)**	40	Sample Depth (mm)	4.0
**Spot size (µm)**	20	Scan mode	Peak hopping
**Scan speed (µm s^{-1})**	40	Integration time (ms)	50 – 55

Table S3. Gene expression analysis from livers of 120 hpf larvae exposed to 1 mM iAs or 2% EtOH. All genes identified in the iAs and EtOH RNA-seq datasets with those significantly overexpressed (red) or downregulated (blue) compared to unexposed siblings highlighted.

Click here to Download Table S3

Table S4. UPR gene expression analysis from livers of 120 hpf larvae exposed to 1 mM iAs or 2% EtOH. All genes identified in the iAs and EtOH RNA-seq datasets with those significantly overexpressed or downregulated compared to unexposed siblings in one or both datasets.

Click here to Download Table S4

Table S5. Number of larvae analyzed for steatosis

Click here to Download Table S5

Table S6. qRT-PCR primer sequences.

Primer Name	Forward Sequence (5' → 3')	Reverse Primer (5' → 3')
**zrplp0**	CTGAACATCTCGCCCTTCTC	TAGCCGATCTGCAGACACAC
**zbip**	AAGAGGCCGAAGAGAAGGAC	AGCAGCAGAGCCTCGAAATA
**zchop**	AAGGAAAGTGCAGGAGCTGA	TCACGCTCTCCACAAGAAGA
**zdnajc3**	TCCCATGGATCCTGAGAGTC	CTCCTGTGTGTGAGGGGTCT
**zedem1**	ATCCAAAGAAGATCGCATGG	TCTCTCCCTGAAACGCTGAT
**zatf4**	TTAGCGATTGCTCCGATAGC	GCTGCGGTTTTATTCTGCTC
**zatf6**	CTGTGGTGAAACCTCCACCT	CATGGTGACCACAGGAGATG
**zxbp1s**	TGTTGCGAGACAAGACGA	CCTGCACCTGCTGCGGACT
**zxbp1t**	GGGTTGGATACCTTGGAAA	AGGGCCAGGGCTGTGAGTA
**zas3mt**	ACTTTATGGGGCGAGTGCCT	AGAGTCGGTATGTGGCAGAC

Supplemental Figure 1

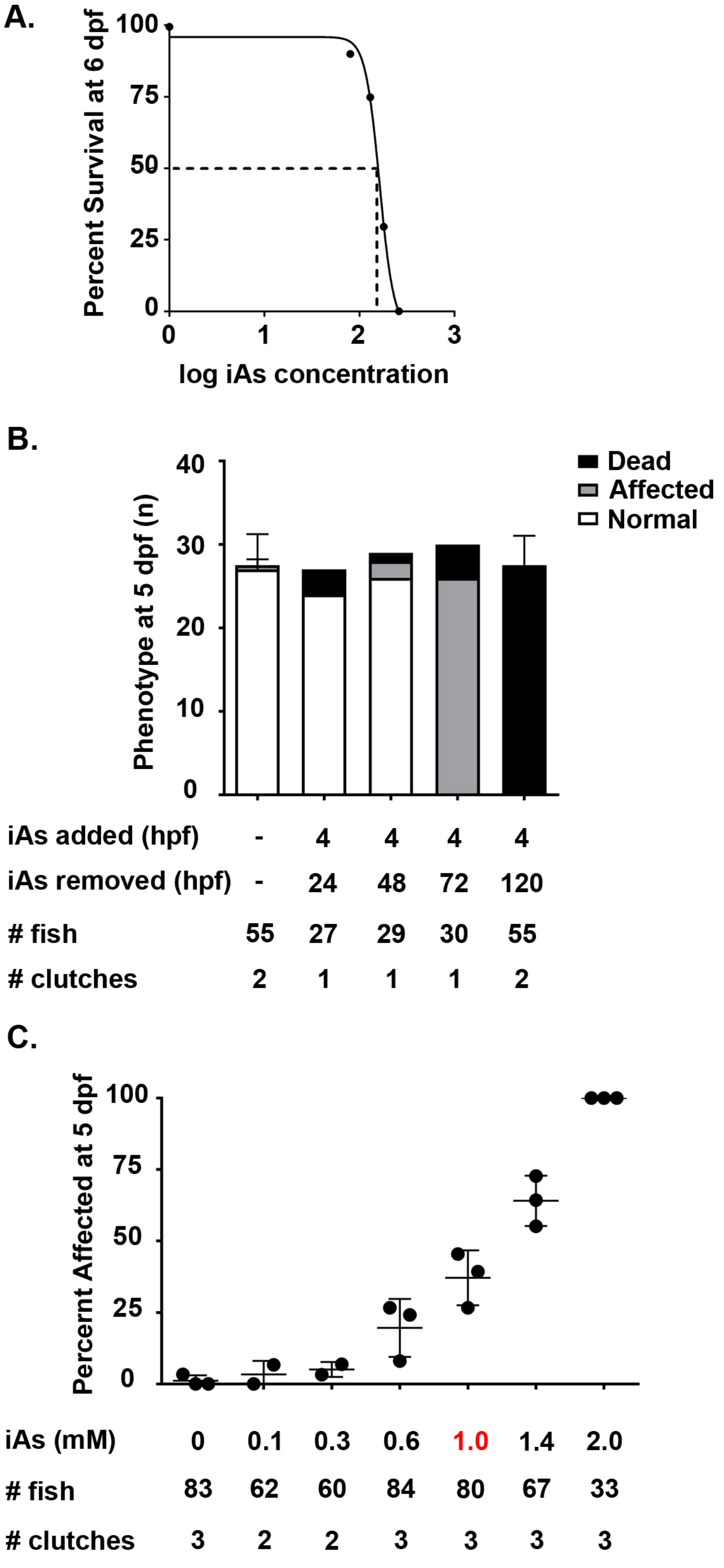

Supplemental Figure 1. Early-life exposure to iAs causes toxicity in zebrafish. A) Calculation of LC_{50} at 6 dpf. B) Time course of removal of 2.0 mM iAs. C) The proportion of surviving embryos at 5 dpf that were affected increased with increasing concentrations of iAs (n = 2 clutches exposed to 0.1 mM or 0.3 mM, n = 3 clutches exposed to 0.6 mM, 1.0 mM, or 1.4 mM, > 30 fish exposed per treatment condition, Table S3). Data from individual clutches presented in Figure 1C.

Supplemental Figure 2

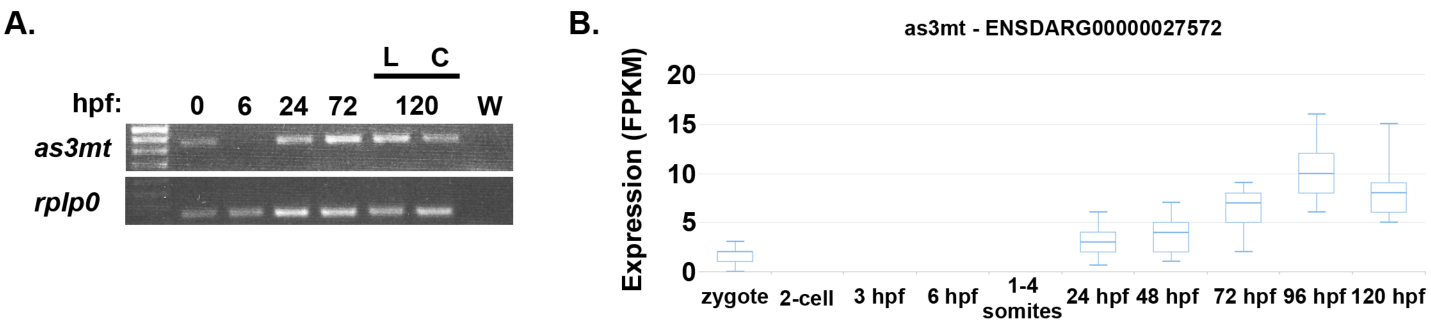

Supplemental Figure 2. Zebrafish embryos and larvae express the trivalent arsenic specific methyltransferase. A) Expression of the as3mt transcript is dynamic during zebrafish development, as determined by reverse-transcription polymerase chain reaction. zas3mt is maternally provided and zygotic expression begins by 24 hpf. Expression is enriched in the liver at 120 hpf. B) Expression of *as3mt* during the first five days of zebrafish development was mined from Array Express.

Disease Models & Mechanisms • Supplementary information

Supplemental Figure 3

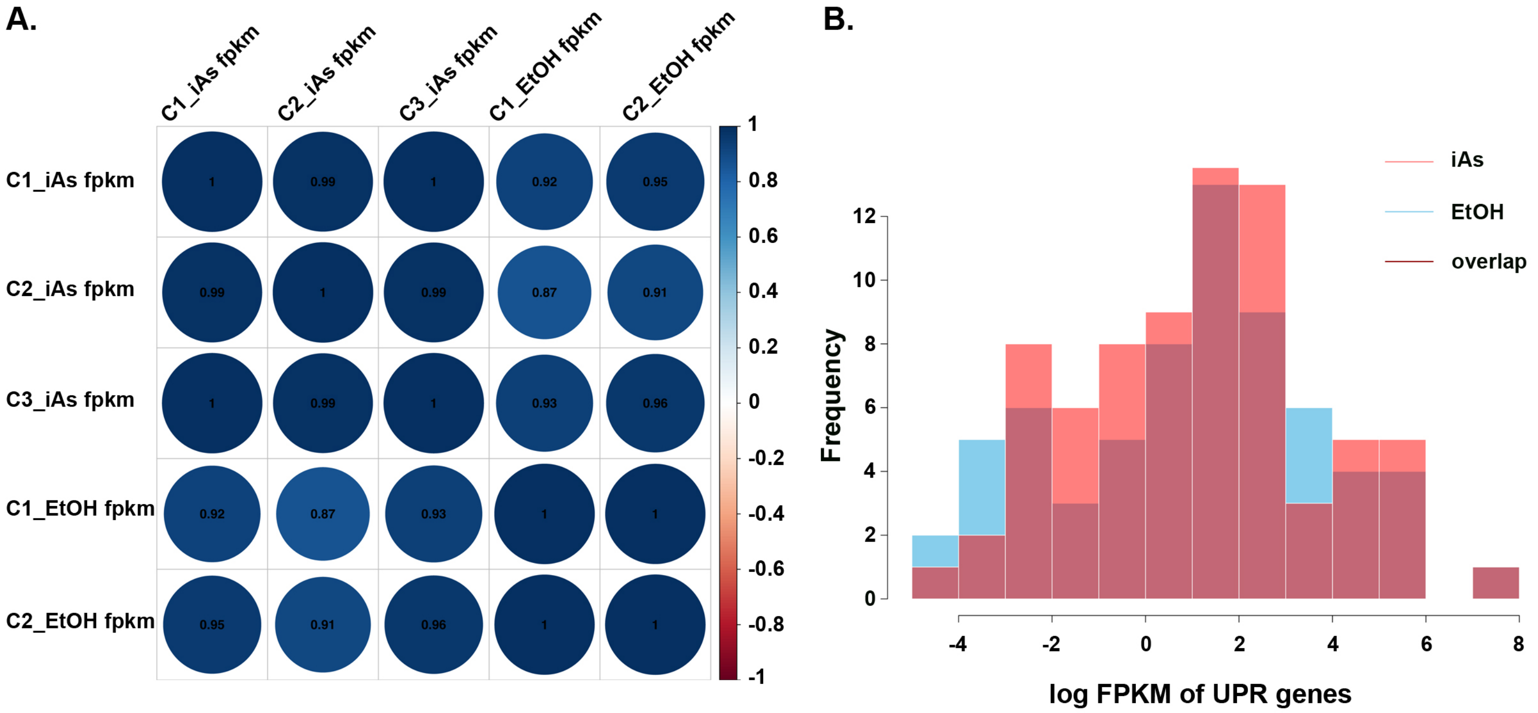

Supplemental Figure 3. Comparison of iAs and ethanol RNAseq datasets. A) Correlation between control samples in iAs and ethanol RNAseq show high similarity in gene expression between these two datasets. B) Expression levels of UPR genes are normally and uniformly distributed in iAs and ethanol RNAseq datasets.

Disease Models & Mechanisms • Supplementary information

Supplemental Figure 4

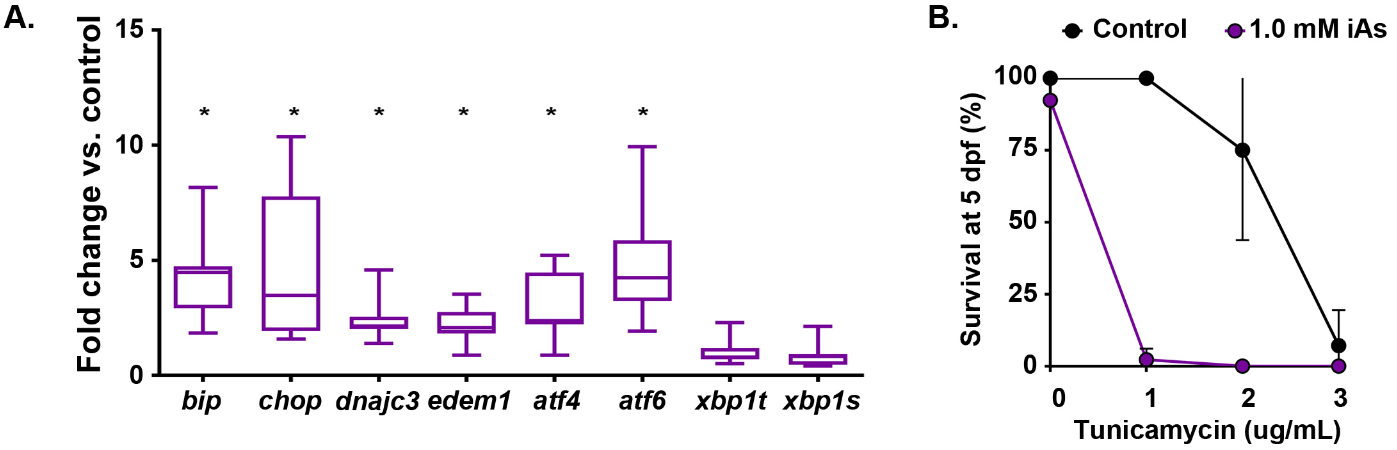

Supplemental Figure 4. Exposure to iAs induces the UPR. A) Quantitative real-time polymerase chain reaction data from liver cDNA. Statistical significance was determined by unpaired, 2-tailed Student’s *t* test (n = 9 clutches, $p < 0.01$). Data are presented as mean, with minimum to maximum. B) Survival of zebrafish larvae at 120 hpf. Zebrafish larvae were exposed to iAs and a dose curve of tunicamycin (0, 1, 2, 3 µg/mL). Survival was assessed at 120 hpf. UPR = unfolded protein response; hpf = hours post fertilization; iAs = inorganic arsenic.

Disease Models & Mechanisms • Supplementary information
