# Supplementary material for: Inorganic arsenic causes fatty liver and interacts with ethanol to cause alcoholic liver disease in zebrafish
Source: Dis Model Mech. 2018 Feb 1;11(2):dmm031575. doi: 10.1242/dmm.031575 (PMC5894941; doi:10.1242/dmm.031575)
Supplement: First Person interview [file dmm-11-031575-s2.pdf]

© 2018. Published by The Company of Biologists Ltd | Disease Models & Mechanisms (2018): doi:10.1242/dmm.031575: Supplementary information

FIRST PERSON

First person – Kathryn Bambino

First Person is a series of interviews with the first authors of a selection of papers published in Disease Models & Mechanisms, helping early-career researchers promote themselves alongside their papers. Kathryn Bambino is first author on 'Inorganic arsenic causes fatty liver and interacts with ethanol to cause alcoholic liver disease in zebrafish', published in DMM. Kathryn is a Postdoctoral Fellow in the lab of Kirsten C. Sadler (NYU Abu Dhabi) and Jaime Chu at Icahn School of Medicine at Mount Sinai, New York, USA, investigating the contribution of environmental modifiers to liver disease.

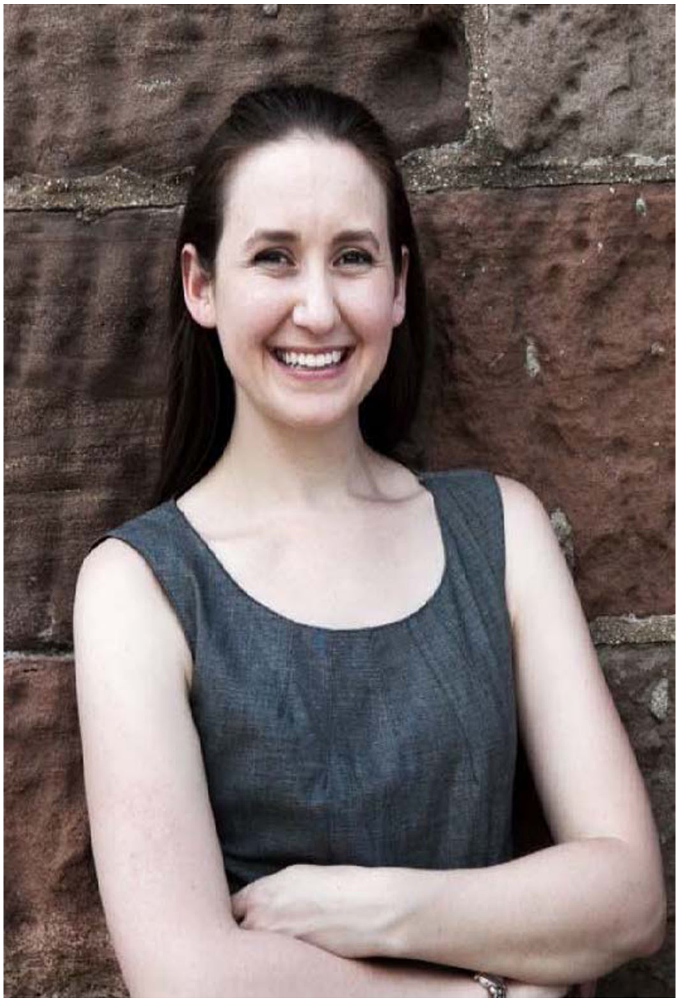

Kathryn Bambino

How would you explain the main findings of your paper to non-scientific family and friends?

Arsenic is a common toxicant found in the environment. Our study uses zebrafish as an experimental model, and we show that arsenic exposure interacts with alcohol exposure to worsen fatty liver disease.

What are the potential implications of these results for your field of research?

This work demonstrates that environmental toxicants can increase the effects of more well-known risk factors to cause disease by activating similar cellular processes.

> "In order to improve the success of early-career scientists, there needs to be more support and substantial funding mechanisms for senior postdoctoral fellows."

What are the main advantages and drawbacks of the model system you have used as it relates to the disease you are investigating?

Zebrafish are a particularly useful model organism to study both toxicity and liver disease. Liver development and metabolic processes are highly conserved between zebrafish and mammals, and their large reproductive capacity allow for the study of large numbers of individuals. The major drawback of using zebrafish larvae is the inability to measure the long-term effects of exposures. In addition, the route and duration of exposure do not completely mimic typical human exposure, as zebrafish only begin to swallow on the third day after fertilization.

Describe what you think is the most significant challenge impacting your research at this time and how will this be addressed over the next 10 years?

There is a lack of understanding of the pathogenesis underlying environmental modifiers of liver disease. Over the next 10 years, there is a critical need to have better communication and collaboration between the fields of environmental toxicology, basic science research, and clinical disease.

What changes do you think could improve the professional lives of early-career scientists?

In order to improve the success of early-career scientists, there needs to be more support and substantial funding mechanisms for senior postdoctoral fellows. In addition, postdocs and junior faculty are often in their child-bearing years, which present particular challenges for scientists trying to sustain high scientific productivity and compete for scarce faculty positions. I believe this will require institutions and funding agencies to offer 'helping hands' funding for scientists who have responsibilities to young children and aging parents. The majority of postdocs at my institution are international and do not have family nearby to help with childcare. Thus, in order to allow gifted scientists to continue their training, many scientist parents also require access to affordable and safe daycare options.

Kathryn Bambino's contact details: Department of Environmental Medicine and Public Health, Icahn School of Medicine at Mount Sinai, New York, USA.
E-mail: kathryn.bambino@mssm.edu

Disease Models & Mechanisms (2018): doi:10.1242/dmm.031575: Supplementary information

What's next for you?

I will be continuing my postdoctoral research on alcoholic liver disease and environmental health as I apply for independent funding and search for academic positions. My goal is to work to form more seamless collaborations between epidemiologists and public health researchers with basic scientists. I believe a transdisciplinary approach will be necessary to more completely understand the associations found in human populations and the mechanistic findings determined in the lab. This will lead to advances in public health by facilitating more appropriate interventions and identifying potential therapeutic opportunities.

Reference

Bambino, K., Zhang, C., Austin, C., Amarasiriwardena, C., Arora, M., Chu, J. and Sadler, K. C. (2018). Inorganic arsenic causes fatty liver and interacts with ethanol to cause alcoholic liver disease in zebrafish. *Dis. Model. Mech.* **11**, doi:10.1242/dmm.031575.
